# Dielectric and Wavefunction Engineering of Electron Spin Lifetime in Colloidal Nanoplatelet Heterostructures

**DOI:** 10.1002/advs.202306518

**Published:** 2024-01-17

**Authors:** Yulu Li, Lifeng Wang, Dongmei Xiang, Jingyi Zhu, Kaifeng Wu

**Affiliations:** ^1^ State Key Laboratory of Molecular Reaction Dynamics Dalian Institute of Chemical Physics Chinese Academy of Sciences Dalian Liaoning 116023 China; ^2^ University of the Chinese Academy of Sciences Beijing Hebei 100049 China

**Keywords:** colloidal nanoplatelets, electron spin relaxation, ultrafast spectroscopy, wavefunction engineering

## Abstract

Colloidal semiconductor nanoplatelets (NPLs) have emerged as low‐cost and free‐standing alternates of traditional quantum wells. The giant heavy‐ and light‐hole splitting in NPLs allows for efficient optical spin injection. However, the electron spin lifetimes for prototypical CdSe NPLs are within a few picoseconds, likely limited by strong electron‐hole exchange in these quantum‐ and dielectric‐confined materials. Here how this hurdle can be overcome with engineered NPL‐heterostructures is demonstrated. By constructing type‐I CdSe/ZnS core/shell NPLs, dielectric screening inside the core is strongly enhanced, prolonging the electron spin polarization time (τ_esp_) to over 30 ps (or 60 ps electron spin‐flip time). Alternatively, by growing type‐II CdSe/CdTe core/crown NPLs to spatially separate electron and hole wavefunctions, the electron‐hole exchange is strongly suppressed, resulting in τ_esp_ as long as 300 ps at room temperature. This study not only exemplifies how the well‐established synthetic chemistry of colloidal heterostructures can aid in spin dynamics control but also establishes the feasibility of room‐temperature coherent spin manipulation in colloidal NPLs.

## Graphical abstract

Colloidal nanoplatelets are low‐cost analogs of quantum wells holding promise for spin‐based quantum technologies. The electron spin lifetimes for typical nanoplatelets are limited to a few picoseconds due to strong electron‐hole exchange interaction. Here through constructing type‐I CdSe/ZnS core/shell and type‐II CdSe/CdTe core/crown nanoplatelets to suppress the exchange interaction, an electron spin lifetime as long as 300 picoseconds is achieved at room temperature.

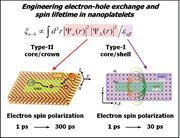

## 1 Introduction

An emerging field in recent years at the intersection of chemistry and physics is to develop low‐cost, solution‐synthesized materials for quantum information science (QIS) and spintronics.^[^ ^1^ ^]^ Exciting opportunities are particularly associated with solution‐processed semiconductor materials such as colloidal nanocrystals^[^ ^2^ ^]^ and metal halide perovskites.^[^ ^3^, ^4^, ^5^, ^6^ ^]^ One reason is that the fundamental principles of spin control have been well established for epitaxially‐grown quantum dots and wells (QDs and QWs),^[^ ^7^, ^8^, ^9^, ^10^, ^11^, ^12^, ^13^, ^14^ ^]^ and it should be straightforward to translate these principles to their solution‐processed counterparts.^[^ ^15^, ^16^ ^]^ Further, the latter is much more flexible in terms of new material/structure design and fabrication.^[^ ^17^ ^]^ A diverse range of core/shell nanocrystals^[^ ^18^ ^]^ and mixed‐dimensionality nano‐heterostructures^[^ ^19^, ^20^, ^21^ ^]^ with no analog in epitaxially‐grown materials have been reported. For example, Au/CdSe core/shell nanocrystals with non‐epitaxial interfaces were synthesized, allowing for spin manipulation controlled by plasmon‐enhanced light‐matter interaction.^[^ ^22^ ^]^ Tunable and even invertible *sp*‐*d* spin exchange interactions have been realized by doping Mn^2+^ into wavefunction‐controllable ZnSe/CdSe core/shell nanocrystals.^[^ ^23^ ^]^

An important family of colloidal materials developed in the past decade is colloidal nanoplatelets (NPLs) with atomically precise thickness.^[^ ^24^, ^25^, ^26^ ^]^ They have unique morphology located at the intermediate regime between epitaxial QWs and QDs, that is, they are not only strongly and precisely confined in their thickness but also weakly confined in their lateral dimensions.^[^ ^27^ ^]^ Meanwhile, unlike colloidal QDs with strongly mixed heavy‐ and light‐hole states that complicate spin polarization injection using optical methods,^[^ ^28^ ^]^ the giant heavy‐light hole splitting on the order of 100 s of meV,^[^ ^25^, ^29^ ^]^ enabled by their highly anisotropic confinement potential, greatly simplifies spin polarization injection by using circularly‐polarized photons.^[^ ^30^, ^31^ ^]^ This efficient spin initialization forms the basis for the following spin manipulation and readout steps for quantum information processing. For efficient spin manipulation, it is essential to understand and control spin relaxation dynamics in these NPLs. While long spin relaxation lifetimes reaching nanoseconds have been reported for CdSe‐based NPLs at cryogenic temperatures^[^ ^32^, ^33^, ^34^, ^35^ ^]^ or even room temperature,^[^ ^36^ ^]^ these lifetimes are mostly contributed by resident carriers or trapped carriers. By contrast, direct measurement of band‐edge electron spin polarization decay using circularly‐polarized femtosecond transient absorption (TA) revealed lifetimes of only a few picoseconds, which are likely limited by strong electron‐hole exchange interaction due to the extreme confinement in the thickness dimension.^[^ ^30^ ^]^

In general, the exchange coupling can be expressed as: ξ_*e* − *h*_ ∝ ∫*d* ^3^ *r*|Ψ_*e*_(*r*)|^2^|Ψ_*h*_(*r*)|^2^/ε_*eff*_, where Ψ_*e*(*h*)_(*r*) is the electron (hole) wavefunction and ε_*eff*_ is the effective dielectric constant. The strong quantum confinement in the thickness and the finite lateral size lead to strong overlap of Ψ_*e*_(*r*) and Ψ_*h*_(*r*) in space. Moreover, these atomically‐thin NPLs are surrounded by organic ligands and solvents of low dielectric constants. The strong leakage of electric‐field lines into the environment strongly reduces ε_*eff*_ inside the NPLs. On the basis of this simple analysis, the roadmap to engineer ξ_*e* − *h*_ becomes clear: one can either reduce the spatial overlap of Ψ_*e*_(*r*) and Ψ_*h*_(*r*)^[^ ^37^, ^38^ ^]^ or increase ε_*eff*_ to decrease ξ_*e* − *h*_ and to prolong electron spin lifetime in colloidal NPLs. Here we illustrate how both strategies can be implemented by the synthetic chemistry of colloidal NPL‐heterostructures. We synthesized type‐I CdSe/ZnS core/shell NPLs, in which the ZnS shell enhances ε_*eff*_ inside the core, and type‐II CdSe/CdTe core/crown NPLs, in which Ψ_*e*_(*r*) and Ψ_*h*_(*r*) are nearly completely separated in space. These strategies allow us to achieve band‐edge electron spin polarization with a lifetime as long as 300 ps at room temperature.

## 2 Results and Discussion

### 2.1 Characterization of CdSe/ZnS Core/Shell NPLs

**Figure 1:**
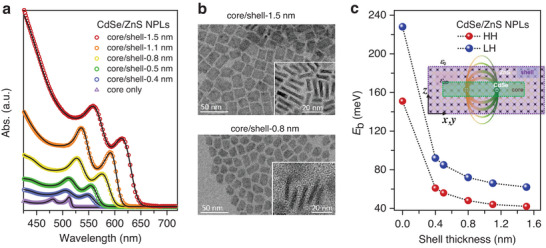
a) UV–Vis absorption spectra of core‐only CdSe NPLs (purple triangles) and CdSe/ZnS core/shell NPLs of varying shell thicknesses (colored circles) dispersed in hexane. The corresponding black lines are the spectral fits. The core/shell samples are labeled by their shell thicknesses. b) Representative TEM images of the samples of core/shell‐0.8 nm (bottom) and core/shell‐1.5 nm (top). c) HH (red balls) and LH (blue balls) exciton binding energies (*E*_b_) plotted as a function of shell thickness. Inset is a scheme showing how electron‐hole interaction inside the core is screened by the coated shell.

To construct the NPL‐heterostructures, we first synthesized CdSe NPLs composed of five atomic layers of Cd and four atomic layers of Se with a thickness of ≈1.4 nm (called four‐monolayer by convention) as the starting core; see Supporting Information for details.^[^ ^25^, ^39^ ^]^ **Figure** **1**a shows the absorption spectrum of CdSe NPLs dispersed in hexane, which displays very sharp excitonic absorption peaks associated with transitions from *n* = 1 heavy‐hole (HH) and light‐hole (LH) to *n* = 1 electron level (E1), at 512 and 480 nm, respectively. We then grew the increasing number of ZnS layers onto the core NPLs using a hot‐injection shell growth method (see Supporting Information).^[^ ^40^ ^]^ The growth takes place in both thickness and lateral dimensions. As a result, the 15 × 10 nm^2^ rectangular CdSe cores are progressively enlarged to 20 × 15 nm^2^ rectangular core/shells with a thickness of 4.5 nm; see transmission electron microscopy (TEM) images and size information in Figure S1 (Supporting Information). Representative TEM images of the samples of core/shell‐0.8 nm and core/shell‐1.5 nm (labeled by their shell thicknesses) are shown in Figure 1b, from the insets of which the enlarged NPL thickness with increasing shell layers can be clearly visualized. However, it should be noted that, unlike the thickness of the CdSe core, the thickness of the ZnS shell is not controlled at an atomically precise level. Consequently, the HH and LH transition peaks are significantly broadened (Figure 1a), consistent with a heterogeneity of the shell thickness.

The gradual redshift of both HH and LH exciton transitions within increasing shell thickness is a consequence of both wavefunction delocalization (i.e., relaxed quantum confinement) and core/shell interfacial biaxial strain, as discussed previously.^[^ ^41^ ^]^ To elaborate on the latter, a large mismatch between the lattice constants of zinc‐blende CdSe (≈0.605 nm) and ZnS (≈0.532 nm) leads to a lateral contraction of the CdSe core sand hence a corresponding expansion in its thickness.^[^ ^42^ ^]^ Screening of Coulomb interactions inside the CdSe core by the ZnS shell might also induce spectral shift, but this shift should be very weak considering that screening effects on exciton binding energy and quasi‐particle bandgap tend to cancel most parts of each other.^[^ ^43^, ^44^ ^]^

Calculating the exciton binding energy (*E*_b_) in the presence of dielectric contrast effect, as is the case for colloidal NPLs, requires high‐level theory^[^ ^45^ ^]^ beyond the scope of the current experimental study. To semi‐quantitatively evaluate the impact of shell thickness on *E*_b_ inside the core, we perform spectral fits to the absorption spectra of core and core/shell NPLs by decomposing them into the sum of excitonic peaks and continuous bands of HH, LH, and spin‐orbit split‐off (SO) bands.^[^ ^46^, ^47^, ^48^ ^]^ The fitting parameters are tabulated in Table S1 (Supporting Information). It is noted that in the fit the ratio between the *E*_b_ of LH and HH excitons is fixed at 1.51. This is because the heavy hole actually has a lighter in‐plane effective mass of 0.19 *m*_e_, whereas the light hole has a lighter in‐plane effective mass of 0.67*m*_e_.^[^ ^49^ ^]^ Using an isotropic electron mass of 0.18 *m*_e_, the in‐plane reduced masses of LH and HH excitons are calculated to be 0.14 and 0.092 *m*_e_, respectively, the ratio between which (1.51) is also the ratio between their binding energies. Figure 1c plots the *E*_b_ of HH and LH excitons as a function of shell thickness. The obtained value of *E*_b_ in core‐only, four‐monolayer NPLs (151 meV for HH exciton) is in reasonable agreement with previous results obtained also from spectral fitting (e.g., 130 meV in ref,^[^ ^47^ ^]^ and 178 meV in ref. [49]). However, these numbers are in general smaller than experimentally measured binding energies (e.g., ≈270 meV in ref. [50]). Here we obtain binding energies using the same fitting model for NPLs of varying shell thicknesses, which serves as a self‐consistent comparison among our samples. The key information is that both *E*_b_ values are reduced by a factor of ≈3.6 from the core‐only sample to the thickest‐shell sample (core/shell−1.5 nm). This reduction is mainly due to an increase in ε_*eff*_ inside the core, which is expected to also reduce electron‐hole exchange interaction.

### 2.2 Spin Relaxation in CdSe/ZnS Core/Shell NPLs

**Figure 2:**
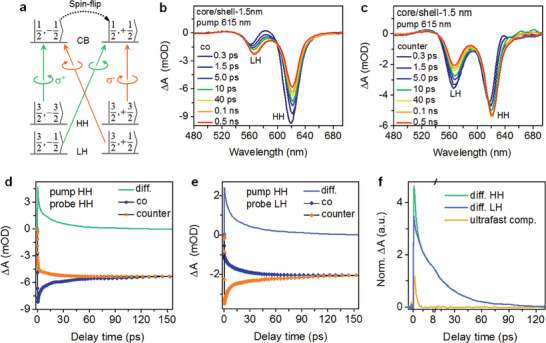
a) Optical selection rules in CdSe colloidal NPLs, for light incident along the surface normal of the NPLs. σ^+^ (green) and σ^−^ (orange) stand for left and right circularly polarized photons carrying angular momenta of +1 and −1, respectively. (b,c) TA spectra of the core/shell‐1.5 nm sample at selected delays following a 615 nm pump (≈ 6.5 µJ cm^−2^), with b) co and c) counter circularly‐polarized pump/probe configurations. d,e) TA kinetics probed at the d) HH and e) LH exciton bleach features with co (navy blue) and counter (orange) pump/probe configurations. The co and counter differences are shown in green and blue lines, respectively. f) The co and counter differential kinetics probed at the HH (green) and LH (blue) bleaches, scaled at their common long‐lived components. The extra ultrafast component in HH kinetics is shown in the dark yellow line.

We applied circularly polarized TA to inject spin polarization and to follow the polarization decay; see SI for details. As illustrated in **Figure** **2**a, a left‐handed σ^+^ photon with energy in resonance with the HH exciton promotes the transition from the |3/2, −3/2> HH to the |1/2, −1/2> electron, whereas a right‐handed σ^−^ photon couples the |3/2, +3/2> HH with the |1/2, +1/2> electron. By using pump and probe photons of the same helicity, TA kinetic traces can be used to follow the decay of the populated polarized electrons and holes. Alternatively, by using pump and probe photons of the opposite helicity, one can monitor the growth of the initially unpopulated spin states. Figure 2b,c presents the TA spectra of the thickest‐shell sample (core/shell−1.5 nm) measured with co‐ and counter‐polarized pump/probe, respectively. The pump wavelength is at 615 nm in resonance with the HH exciton transition. Note the presence of both HH and LH exciton bleaches under this pump is because they share the same electron level.

By monitoring the HH bleach, we find that the signal indeed decays and grows in the cases of co‐ and counter‐polarized pump/probe, respectively (Figure 2d), verifying the injection of spin polarization. However, we find that the initial signal size with a counter‐polarized pump/probe is much higher than zero. This is because the current experiments were performed with hexane‐dispersion of NPLs in which they are randomly oriented. The optical orientation rules in Figure 2a are drawn for light incident along the surface normal of the NPLs. For light incidents along other directions, the selection rules are more complicated. Interestingly, when monitoring the LH exciton bleach, we find that the signal instead grows and decays in the cases of co‐ and counter‐polarized pump/probe (Figure 2e). This can be well rationalized by the spin selection rules in Figure 2a: While the |1/2, −1/2> electron generated by a σ^+^ pump photon blocks the transition from the |3/2, −3/2> HH, it also blocks the transition from the |1/2, +1/2> LH that is coupled to a σ^−^ probe photon.

In spite of the above‐mentioned orientational issue, by taking the difference between the co and counter‐TA data, the dynamics of those successfully injected spins can be probed. Consistent with a previous study on plain CdSe NPLs,^[^ ^30^ ^]^ the differential kinetics probed at the HH exciton bleach clearly reveals a rapid spin polarization decay (Figure 2d; Figure S2, Supporting Information), and that probed at the LH exciton bleach reveals an overall similar but slightly different decay curve (Figure 2e; Figure S2, Supporting Information). By plotting the two polarization decay curves together (Figure 2f), we find that they share identical slow components, but the HH kinetics contain an extra ultrafast decay component. By performing a subtraction between these two, the ultrafast component (within a few ps) can be isolated. The nature of this ultrafast component is unclear–it could be associated with rapid hole spin‐flip,^[^ ^30^, ^51^, ^52^ ^]^ or it could be due to other depolarization mechanisms unique to the HH exciton band that is resonantly excited in our experiments. Here we focus on the common slow components probed at the HH and LH exciton bleaches, which can be largely assigned to electron spin polarization decay as these two exciton bands clearly share the same electron states. The slow components can be fitted to biexponential decays with time constants of 3.7 and 33.9 ps, respectively (Table S2, Supporting Information). The heterogeneous kinetics likely results from the shell thickness distribution. A simple kinetic analysis indicates that the electron spin polarization decay time (*τ*_esp_) is half of the time constant of the elementary electron spin‐flip process.^[^ ^53^ ^]^ Thus, *τ*_esp_ over 30 ps corresponds to an electron spin‐flip time over 60 ps. We further note that, following electron spin depolarization, the electron populations are long‐lived, as evident from the long‐lived plateaus after co and counter TA signals converge (Figure 2d,e). Therefore, at room temperature, the electron‐hole recombination of NPLs is not a factor limiting the carrier spin lifetimes in these systems.

**Figure 3:**
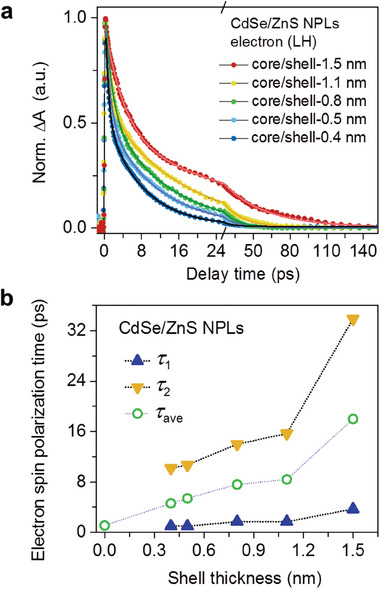
a) Electron spin polarization decays probed at the LH bleach for CdSe/ZnS samples of varying shell thicknesses (colored symbols) and their biexponential fits (colored lines). b) Fast (blue triangles), slow (yellow triangles), and amplitude‐averaged (green circles) time constants of electron spin polarization decay as a function of shell thickness. Note the core‐only sample has only one time component.

In **Figure** **3**a, we plot the kinetics of electron spin polarization decays, probed with the LH bleach, of the core/shell samples with varying shell thicknesses; see their circular‐polarization TA data in Figures S3–S6 (Supporting Information). The electron spin relaxation progressively slows down with increasing shell thickness. By fitting these kinetic traces using biexponential decays, we obtain the time constants of their fast and slow components; see Table S2 (Supporting Information) for fitting parameters. As plotted in Figure 3b, the fast and slow components grow from 1 ps to 3.7 ps, and from 10.2 to 33.9 ps, respectively, with increasing shell thickness. Because the time constants of the fast components are similar to or only slightly longer than the electron spin polarization decay time of 1.1 ps in plain CdSe NPLs of four monolayers,^[^ ^30^ ^]^ we assign the fast component of each sample to a subset of core/shell NPLs with relatively thin or “leaky” shells in the sample, for which the electron‐hole exchange interaction is not effectively screened (i.e., with small ε_*eff*_). On the other hand, the slow component corresponds to the other subset of well‐coated core/shell NPLs with large ε_*eff*_ and strongly screened electron‐hole exchange.

We note that the relaxation of the quantum confinement energy in the CdSe core by the ZnS shell should not be a dominant factor contributing to the prolonged electron spin lifetime. As a reference, for plain CdSe NPLs, *τ*_esp_ only increases from 1.1 to 3.1 ps when the thickness increases from 4 to 6 monolayers which is accompanied by a redshift of the HH exciton peak from 512 to 582 nm.^[^ ^30^ ^]^ By contrast, for the CdSe/ZnS core/shell sample of similar confinement energy (core/shell−0.8 nm), its *τ*_esp_ reaches 14 ps. Additionally, we will make further discussions below (after we present the results on type‐II NPLs) to exclude other possible mechanisms for the prolonging of *τ*_esp_ by the ZnS shells. This allows us to attribute the significantly prolonged *τ*_esp_ in CdSe/ZnS core/shell NPLs mainly to effective screening of the electron‐hole exchange interaction by the inorganic ZnS shell.

The rate of electron spin polarization decay, 1/*τ*_esp_, should scale with the square of exchange coupling (|ξ_*e* − *h*_|^2^), and thus scale inversely with|ε_*eff*_|^2^. The exciton binding energy (*E_b_*) also scales with the inverse of |ε_*eff*_|^2^. Therefore, *τ*_esp_ should scale with the inverse of *E_b_*. As revealed in Figure 1c and Table S1 (Supporting Information), *E*_b_ inside the thickest shell sample (core/shell‐1.5 nm) is reduced by a factor of 3.6 compared to the core‐only sample. This cannot fully explain why *τ*_esp_ is prolonged by a factor of ≈16 from the core‐only sample (1.1 ps) to the thickest‐shell sample (amplitude‐averaged value of 18 ps; Figure 3b). Possible reasons for the discrepancy include that *E*_b_ is determined from an empirical spectral fitting and that the sample heterogeneity complicates the evaluation of both *E*_b_ and *τ*_esp_.

### 2.3 Characterization of CdSe/CdTe Core/Crown NPLs

**Figure 4:**
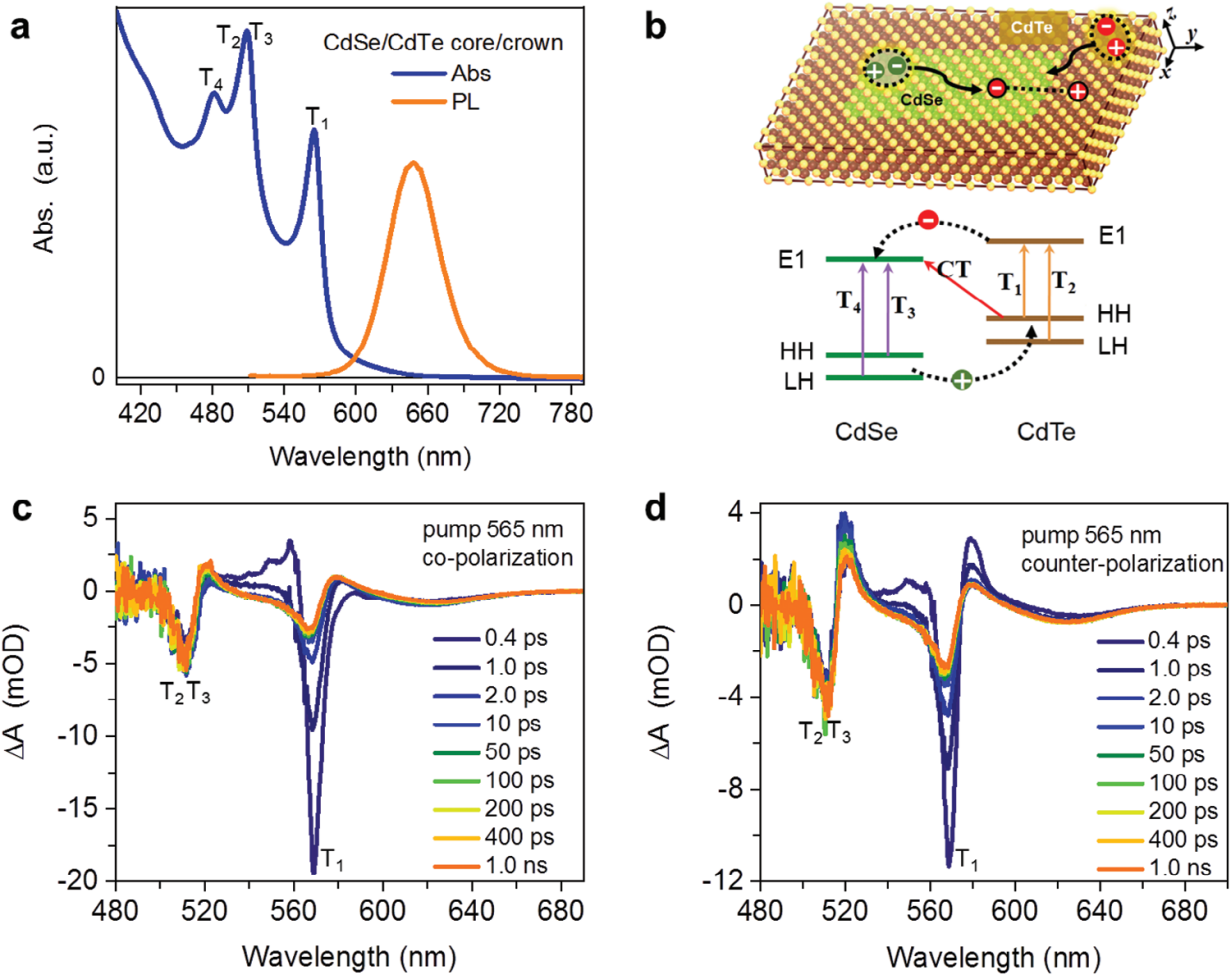
a) UV‐Vis absorption (blue) and PL (orange) spectra of type‐II CdSe/CdTe core/crown NPLs grown from four‐monolayer CdSe NPLs. Several transition features are indicated. b) Schemes showing the structure of a core/crown NPL (top) and the energy levels and transitions (bottom). c,d) TA spectra of CdSe/CdTe core/crown NPLs measured under c) co‐ and d) counter‐polarized pump/probe configurations.

Having established that type‐I CdSe/ZnS core/shell NPLs can indeed prolong *τ*_esp_ by increasing dielectric screening, we further use type‐II CdSe/CdTe heterostructures to elucidate the effect of spatially separating Ψ_*e*_(*r*) and Ψ_*h*_(*r*).^[^ ^37^ ^]^ For this purpose, we synthesized core/crown structures in which CdTe crowns are laterally, and in an iso‐thickness fashion, extended on CdSe NPLs of four monolayers;^[^ ^54^, ^55^, ^56^ ^]^ see Figure S7 (Supporting Information) for details. **Figure** **4**a presents the absorption and PL spectra of the core/crown NPLs in hexane. The HH and LH exciton absorption peaks at 514 nm (T_3_) and 480 nm (T_4_), respectively, from the CdSe cores are preserved, whereas the new absorption feature at 565 nm (T_1_) can be assigned to the HH exciton of the CdTe crowns. The LH exciton of CdTe should be situated at ≈510 nm (T_2_) which accidentally has a nearly complete spectral overlap with the HH exciton of CdSe. The PL spectrum is considerably broadened and redshifted than the exciton absorption features of both CdSe and CdTe. This PL arises from a charge‐transfer (CT) transition between the CB of CdSe and the HH of CdTe (Figure 4b). Nearly complete quenching of both CdSe and CdTe HH exciton emissions indicates that inter‐domain charge separation is extremely rapid and efficient in this system.^[^ ^56^, ^57^ ^]^ In accordance with this PL feature, a CT absorption feature is identified as the weak tail extending to ≈660 nm. The large bandwidth of both absorption and PL of the CT transition, in spite of an atomically precise thickness of the core/crown sample, originates from a strong coupling between the CT exciton and polar phonons, as noted in previous studies.^[^ ^57^, ^58^ ^]^

We applied TA to directly observe rapid inter‐domain charge separation in the core/crown NPLs and to examine whether this process is rapid enough to transfer electron spin polarization from the CdTe crowns to the CdSe cores. To this end, the pump wavelength was chosen at 565 nm, at which wavelength the absorption is dominated by CdTe with only slight contributions from the CT transition. Figure 4 panels c and d present the TA spectra measured under co‐ and counter‐polarized pump/probe configurations, respectively. In general, the bleach features associated with the HH excitons of CdTe (at 565 nm) and CdSe (at 514 nm) can be observed, along with the broad‐band bleach of the CT transition centered at ≈630 nm. The LH exciton bleach of CdSe is spectrally inaccessible in our setup, whereas that of CdTe is covered by the CdSe HH exciton bleach.

**Figure 5:**
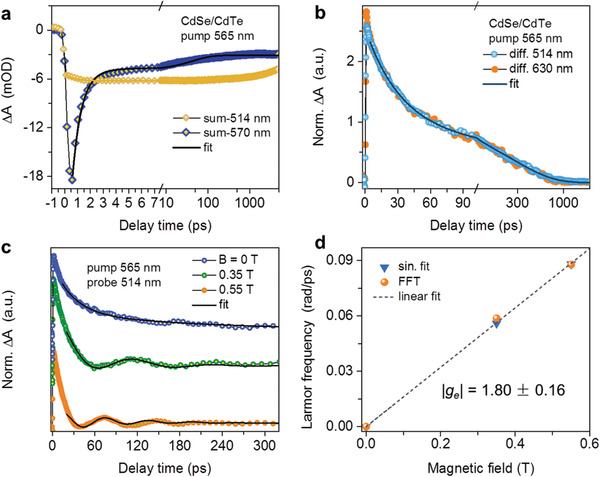
a) Sum of the co and counter TA kinetics probed at the HH bleach of CdTe (570 nm), and at that of CdSe (514 nm). The black solid line is an exponential fit revealing an electron transfer time of 0.7 ps. b) Differences of the co and counter TA kinetics probed at wavelengths near the CdSe HH bleach (blue circles) and near the CT bleach (orange dots). The solid line is a biexponential fit. c) Electron spin precession kinetics about external transversal magnetic fields of 0.55 T (orange circles) and 0.33 T (green circles). The black solid lines are their damped sinusoidal fits. d) Larmor precession frequency (*ω*_L_), obtained from kinetic fits (blue triangles) and from FFT (orange balls), as a function of the field strength, and their linear fit (dashed line).

By taking the sum of the co and counter TA kinetics, we can first ignore the spin polarization information and monitor charge transfer dynamics. **Figure** **5**a shows the sums probed at the HH exciton bleaches of CdTe (570 nm) and CdSe (514 nm). While the decay of the former is clearly identified, the complementary growth of the latter is not observed. This is likely because the growth of the CdSe HH exciton bleach is almost canceled by the decay of the spectrally overlapped CdTe LH exciton bleach. Nevertheless, by fitting the kinetics at the CdTe HH exciton bleach, we find an electron transfer time constant of 0.7 ps from the CB of CdTe to that of CdSe, consistent with the one reported in a previous study.^[^ ^56^ ^]^

### 2.4 Spin Relaxation in CdSe/CdTe Core/Crown NPLs

The sub‐ps inter‐domain electron transfer should suffice to transfer electron spin polarization from CdTe to CdSe before it is lost to depolarization channels. An experimental manifestation is that TA kinetics probed at multiple wavelengths near the CdSe HH bleach as well as near the CT bleach show sizable differences in the amplitudes of co and counter TA signals (Figure S8, Supporting Information). Kinetics of electron spin polarization can thus be isolated by taking the differences, as presented in Figure 5b. These kinetic traces are well consistent with each other and can be fitted to biexponential decays with fast and slow time constants of 32 and 287 ps, respectively. The presence of two distinct time constants is likely also associated with the sample heterogeneity, as is for CdSe/ZnS core/shell NPLs.

However, while it is straightforward to argue that the variation in shell thickness/coverage affects ε_*eff*_ and hence the electron‐hole exchange interaction in CdSe/ZnS core/shell NPLs, the specific source of heterogeneity in CdSe/CdTe core/crown NPLs is not clear, as they are atomically precise in thickness. One possible source of heterogeneity is the spatial distribution of surface defects in the CdTe crowns. In a subset of NPLs, after the electron is transferred to CdSe, the hole is localized at the defect site near the core/crown interface, and hence the electron‐hole exchange interaction is still relatively strong, causing relatively fast electron spin relaxation (32 ps). By contrast, if the hole is localized far from the interface, Ψ_*e*_(*r*) and Ψ_*h*_(*r*) is almost completely separated, which results in a diminishing exchange interaction and thus a slow electron spin relaxation (287 ps). Additionally, the interface heterogeneity could also be a reason, as small changes at the interface, such as interfacial alloying or strain,^[^ ^59^, ^60^ ^]^ could also modify the electron‐hole wavefunction overlap, and hence the strength of exchange interaction.

In a previous study on CdTe/CdSe core/shell QDs using a cross‐polarized heterodyne third‐order transient grating technique,^[^ ^37^ ^]^ Scholes et al. reported that the hole spin relaxation time can be prolonged from ≈0.3 to 10 ps, also by reducing the electron‐hole exchange through wavefunction separation. They have shown in their prior works^[^ ^51^, ^52^ ^]^ that this technique is mostly sensitive to hole spin relaxation within the excitonic fine structures. By contrast, herein we use circularly polarized, broadband TA to study spin relaxation, the advantage of which is that we can clearly identify the state‐filling signals induced by a specific type of carrier. For example, following the rapid electron transfer from the CB of CdTe to that of CdSe, the bleach we probed at 514 nm is deterministically associated with the electrons in CdSe, thereby allowing us to attribute the observed spin lifetime to the electrons. In principle, after electron‐hole separation, the hole spin lifetime could also be prolonged, which, however, is not easy to capture with our technique.

The electron spin relaxation on the timescale of 100 s of ps in our CdSe/CdTe NPLs is likely induced by the weak spin‐orbit coupling in the CB (e.g., the Elliott‐Yafet mechanism) and the hyperfine interaction with fluctuating nuclear spins.^[^ ^36^, ^61^ ^]^ In principle, it is possible to suppress the Elliott‐Yafet relaxation by lowering the temperature. At present, our CdSe/CdTe NPLs tend to degrade rapidly after their deposition onto solid‐state films, likely induced by the low stability of the CdTe crowns. We are currently working on improving the chemical and photostability of these NPLs to enable low‐temperature experiments.

The electron spin polarization lifetime approaching 300 ps allows us to observe spin precession about a weak external magnetic field (*B_z_*). *B_z_* is applied perpendicularly to the laser beams, which corresponds to the Voigt geometry. Figure 5c presents the spin procession dynamics under *B_z_* of 0.35 and 0.55 T. The magnetic field dependence of the coherent oscillation also substantiates that it is due to Larmor precession rather than other origins such as coherent phonons.^[^ ^62^ ^]^ The Larmor precession frequency is: *ω*_L_ = *g_e_µ*_B_ *B_z_*/ℏ, where *g_e_* is the electron g‐factor in CdSe, *µ*_B_ the Bohr magneton, and ℏ the reduced Planck constant. By either fitting the kinetic traces in Figure 5c with damped sinusoidal functions or performing fast Fourier transformation (FFT) on the kinetics, we obtain excellent linear scaling between *ω*_L_ and *B_z_* (Figure 5d). The absolute value of the electron g‐factor is |*g_e_* | = 1.80 ± 0.16. This value is consistent with the electron g‐factors reported for CdSe‐based materials of similar confinement energy.^[^ ^2^, ^61^ ^]^ Thus, the spin precession experiment further evidences that the electron spin polarization is efficiently transferred from initially excited CdTe crowns to CdSe cores.

As briefly mentioned above, combining the results from type‐I CdSe/ZnS core/shell NPLs and type‐II CdSe/CdTe core/crown NPLs provides further insight into the mechanisms of electron spin depolarization in these NPLs. Previous studies performed under cryogenic temperatures have revealed the importance of surface dangling bond (SDB) spins in controlling the exciton/carrier spin lifetimes in NPLs.^[^ ^32^ ^]^ Therefore, the elongation of τ_esp_ with increasing shell thickness in CdSe/ZnS core/shell NPLs could originate from the isolation of core spins from SDB spins.^[^ ^31^ ^]^ However, in type‐II CdSe/CdTe NPLs, the CdTe crowns are laterally extended from the central CdSe cores, which do not isolate the core spins from SDB spins but only separate electron and hole spins into distinct spatial domains. The remarkably long τ_esp_ of ≈300 ps thus indicates that, at room temperature and within the timescale of hundreds of ps, the electron‐hole exchange interaction is the limiting factor to τ_esp_. Nevertheless, we do not exclude the importance of SDB spins, as they could indeed play a major role under cryogenic temperatures and on timescales longer than ns.

## 3 Conclusion

We demonstrated the strategies of using NPL‐heterostructures to engineer the electron‐hole exchange interaction and thus the electron spin polarization lifetime in these colloidal materials. Starting from the fundamental expression of exchange coupling strength, we designed two types of NPL‐heterostructures, type‐I CdSe/ZnS core/shell NPLs and type‐II CdSe/CdTe core/crown NPLs, in which the effective screening is enhanced and the electron and hole wavefunctions are spatially separated, respectively. In contrast to the electron spin polarization time (τ_esp_) of only ≈1 ps in plain CdSe NPLs, in CdSe/ZnS core/shell NPLs τ_esp_ exceeded 30 ps. Even more exciting is that τ_esp_ reached 300 ps in CdSe/CdTe core/crown NPLs, enabled by nearly complete separation of electron and hole wavefunctions. Further, for this system, we have observed coherent electron spin precession about an external magnetic field. Since all these measurements were performed at room temperature, the results herein suggest the possibility of room‐temperature coherent quantum‐state control of electron spins in colloidal NPLs.

## Conflict of Interest

The authors declare no conflict of interest.

## Supporting information

Supporting Information

## Data Availability

The data that support the findings of this study are available from the corresponding author upon reasonable request.
