# Supplementary material for: Dielectric and Wavefunction Engineering of Electron Spin Lifetime in Colloidal Nanoplatelet Heterostructures
Source: Adv Sci (Weinh). 2024 Jan 17;11(12):2306518. doi: 10.1002/advs.202306518 (PMC10966543; doi:10.1002/advs.202306518)
Supplement: Supplementary file 1 — Supporting Information [file ADVS-11-2306518-s001.pdf]

ADVANCED SCIENCE
Open Access

Supporting Information

for *Adv. Sci.*, DOI 10.1002/advs.202306518

Dielectric and Wavefunction Engineering of Electron Spin Lifetime in Colloidal Nanoplatelet Heterostructures

Yulu Li, Lifeng Wang, Dongmei Xiang, Jingyi Zhu and Kaifeng Wu**

Supporting Information for:

Dielectric and wavefunction engineering of electron spin lifetime in colloidal nanoplatelet heterostructures

Yulu Li, [1†] *Lifeng Wang,* [1, 2 †] *Dongmei Xiang,* [1] *Jingyi Zhu* [1*] *and Kaifeng Wu*[1*]

[1] State Key Laboratory of Molecular Reaction Dynamics, Dalian Institute of Chemical Physics, Chinese Academy of Sciences, Dalian, Liaoning 116023, China

[2] University of the Chinese Academy of Sciences, Beijing 100049, China

Experimental Methods

Sample preparations

Preparation of cadmium myristate. Cadmium myristate was synthesized by following the recipe reported in the literature with slight modifications.[1] Sodium myristate (3.13 g) was dissolved in 250 mL of methanol under strong stirring. Cadmium nitrate tetrahydrate (1.23 g) was dissolved in 40 mL of methanol and added dropwise to the sodium myristate solution. After addition, the reaction mixture was stirred for two hours. The whitish product was washed with methanol three times for the removal of excess precursors. Subsequently, the final product was collected by filtration and dried under vacuum.

Synthesis of four monolayer (4 ML) CdSe NPLs. 4 ML CdSe NPLs were synthesized according to the literature, with slight modifications.[1, 2] For a typical synthesis, 170 mg of cadmium myristate, 12 mg of Se, and 30 mL of octadecene (ODE) were loaded into a 50 mL three-neck flask. The solution was degassed and stirred at 95 ^{o}C under vacuum for an hour to evaporate volatile solvents and dissolve

the cadmium myristate completely. The heater was then set to 240 ^{o}C. When the temperature reached 100 ^{o}C, the flask was switched from vacuum to nitrogen gas. As the temperature reached 200 ^{o}C, the color of the solution became orange, and at this stage 40 mg of cadmium acetate dihydrate ($Cd(Ac)_2 \cdot 2H_2O$) was introduced swiftly into the reaction. After the growth of CdSe NPLs at 240 ^{o}C for around 10 minutes, 1 mL of oleic acid (OA) was injected when the temperature of the solution was decreased to 160 ^{o}C using air gun. 15 mL of Hexane was injected when the temperature is decreased to room temperature. The solution was centrifuged for 10 min at 3000 rpm, and the supernatant was transferred another centrifuge tube. This solution was centrifuged at 7800 rpm for 10 min, and the precipitates were dissolved and stored in toluene for further use.

Synthesis of CdSe/ZnS core/shell NPLs. CdSe/ZnS core/shell NPLs were synthesized following reported procedures with slight modifications.[3] In a typical reaction, 0.2 mmol zinc acetate, 7 mL 4 ML CdSe NPLs (with optical density of 1.6 at 512 nm in 1 mm cuvette), 0.5 mL OA, and 5 mL ODE were added to a 50 mL three-neck flask. The solution was stirred under vacuum at room temperature for an hour to evaporate hexane. Subsequently, the mixture was heated up to 90 ^{o}C and kept for 30 min to completely remove water and/or other remaining volatile solvents. After the degassing step, 0.5 mL oleylamine (OLAm) was added into the solution under nitrogen flow. The reaction was then kept for 10 min and the temperature was set to 300 ^{o}C. An octanethiol-ODE solution (87.5 μL octanethiol in 5 mL ODE) was prepared in glove box and injected into the reaction at 170 ^{o}C using a syringe pump with a speed of 8.85 mL/h. When the temperature reached 250 ^{o}C, the injection speed of the octanethiol-ODE solution was changed to 3.54 mL/h. The reaction was kept at 300 ^{o}C for 60 min, after which it was quenched by injection of 5 mL ODE and an ice-water bath. The product was washed with 20 mL ethanol and centrifuged at 5500 rpm for 3 min. The precipitated final product was dispersed in hexane for optical characterization. CdSe/ZnS core/shell NPLs with different shell thicknesses were

obtained by changing the amount of OA and OLAm. By varying the amount of ligand from 0.02 to 1 mL, the shell thickness can be increased from 0.4 nm to 1.5 nm, as shown in Figure S1.

Synthesis of CdSe/CdTe core/crown NPLs. The CdSe/CdTe core/crown NPLs were synthesized following procedures reported in literature with slight modifications.[2] 3 mL CdSe 4 ML NPLs (with optical density of 1 at 512 nm in 1 mm cuvette), 80 mg of $Cd(Ac)_2{\cdot}2H_2O$, and 0.05 mL of OA was dissolved in 10 mL of ODE and degassed under vacuum for 30 min at 100 ^oC, and then heated to 190 ^oC under nitrogen flow. The tellurium (Te) precursor was made by string a mixture of 1.7 mg Te powder, 0.4 mL Trioctylphosphine (TOP), and 5 mL ODE overnight in a glovebox. The Te precursor was then slowly injected using a syringe pump at a rate of 1 mL/hour. The reaction was quenched after ~1 hour and the product was washed by precipitation with acetone followed by centrifugation, and finally dispersed in hexane for optical characterizations.

Transient absorption

The femtosecond pump-probe TA measurements were based on a Pharos laser (1030 nm,100 kHz, 230 fs pulse-duration; Light conversion). One part of the Pharos output was used to pump an optical parametric amplifier (OPA; TOPAS) to generate the wavelength-tunable excitation pulses, while the other was sent through a delay stage and attenuated with a neutral density filter and focused into a 1 cm thick sapphire window to generate a white light continuum (WLC) used as the probe beam. The pump and probe beams were focused and overlapped onto the sample. Circularly polarized pump and probe pulse were generated separately by two sets of broadband polarizing beam splitter cubes (400-700 nm, Thorlabs) and quarter-wave plates

(350-850 nm, Thorlabs). Transverse magnetic fields were provided by an electromagnet (EM3; Beijing Jinzhengmao Technology Co.).

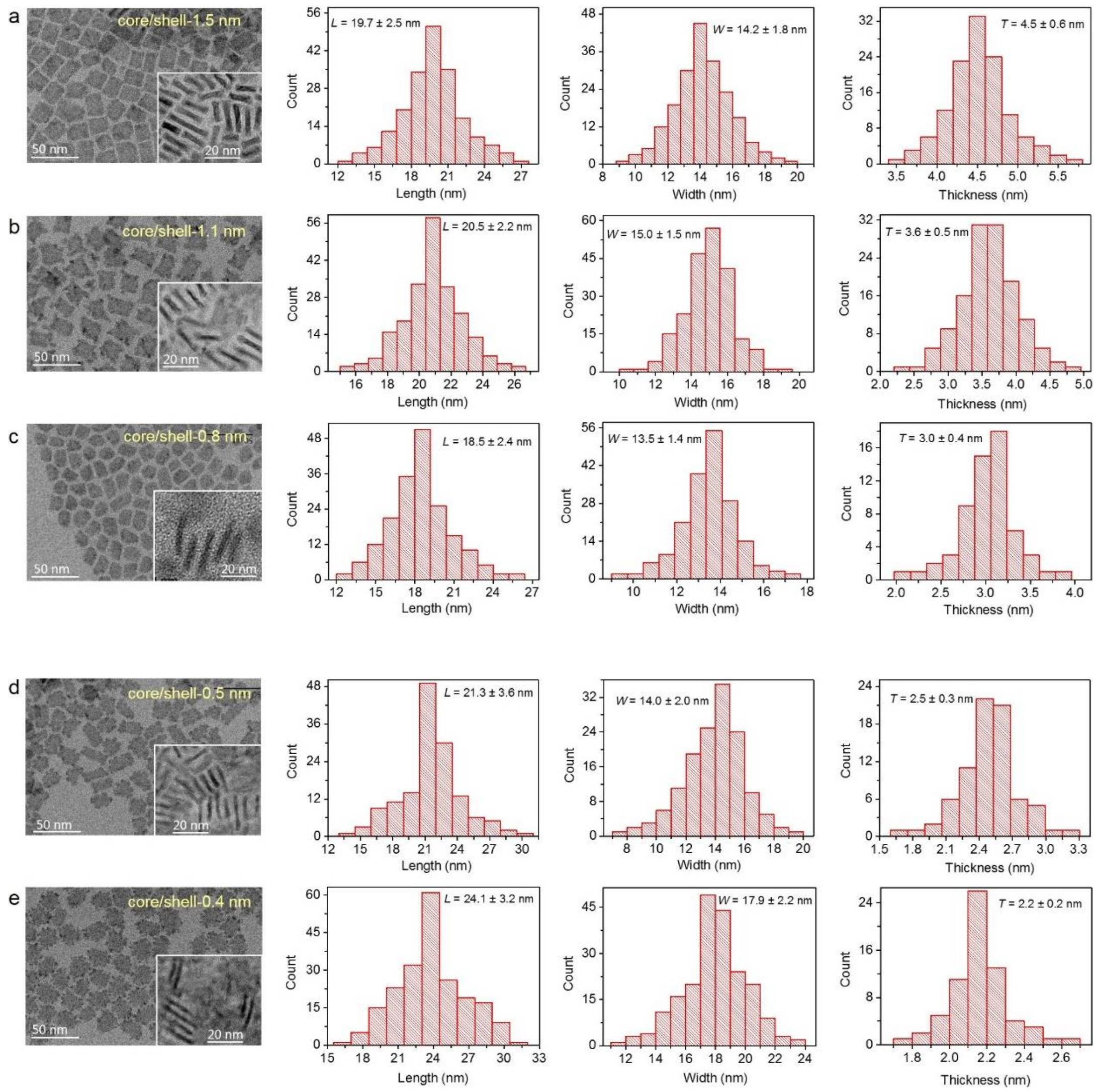

Figure S1. TEM images of CdSe/ZnS core/shell colloidal NPLs (left) and the corresponding histograms for the NPLs' length, width and thickness (right). (a) core/shell-1.5 nm, (b) core/shell-1.1 nm, (c) core/shell-0.8 nm, (d) core/shell-0.5 nm, (e) core/shell-0.4 nm.

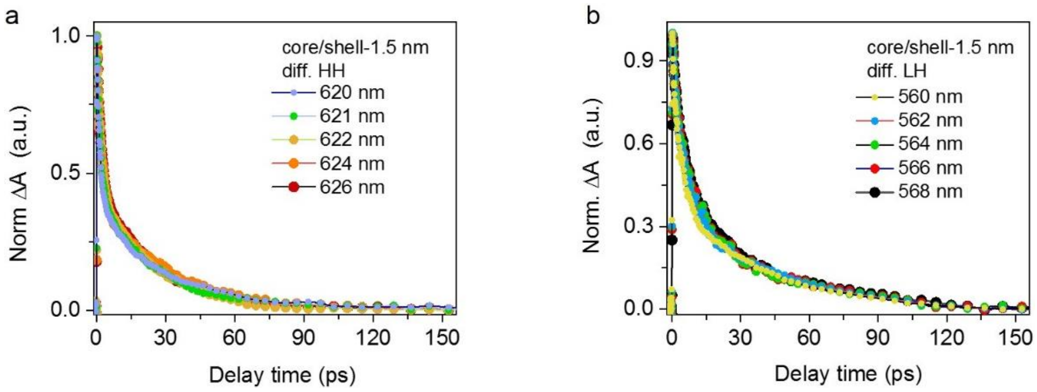

Figure S2. The differential kinetics probed at multiple wavelengths, (a) near the HH exciton bleach, and (b) near the LH exciton bleach, for the CdSe/ZnS core/shell-1.5 nm sample.

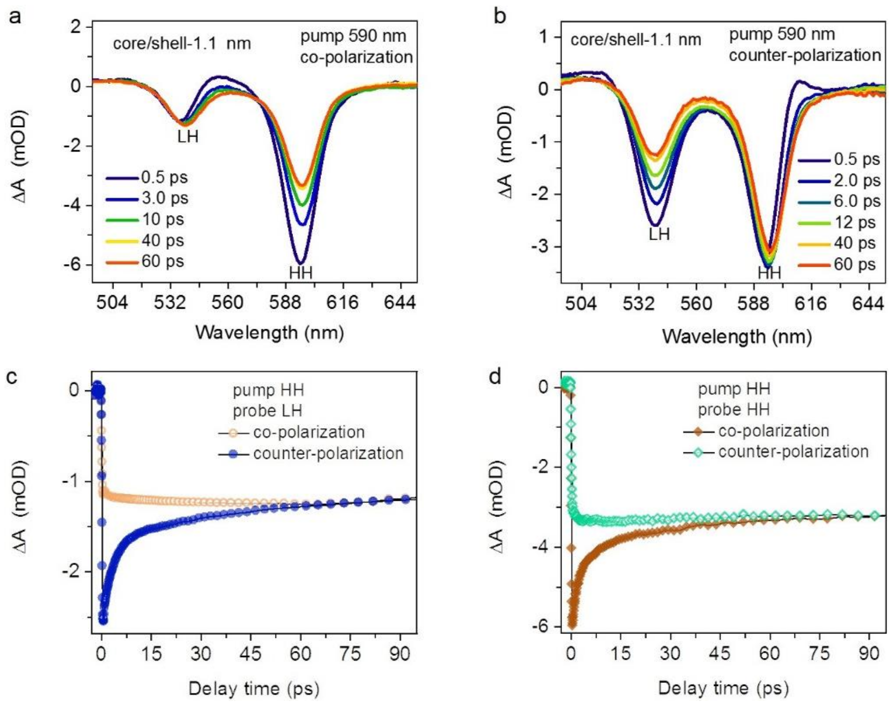

Figure S3. TA spectra of CdSe/ZnS core/shell-1.1 nm NPLs measured with (a) co- and (b) counter-polarized pump/probe configurations. TA kinetics of CdSe/ZnS NPLs measured with co- and counter-polarized pump/probe configurations probed at (c) HH (590 nm) and (d) LH (536 nm) exciton bleach features.

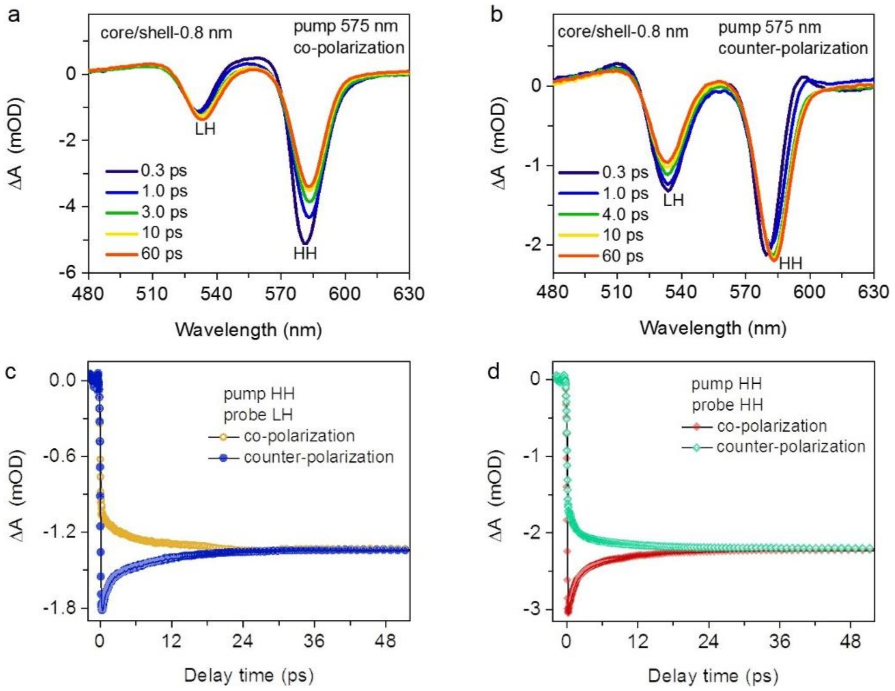

Figure S4. TA spectra of CdSe/ZnS core/shell-0.8 nm NPLs measured with (a) co- and (b) counter-polarized pump/probe configurations. TA kinetics of CdSe/ZnS NPLs measured with co- and counter-polarized pump/probe configurations probed at (c) HH (575 nm) and (d) LH (526 nm) exciton bleach features.

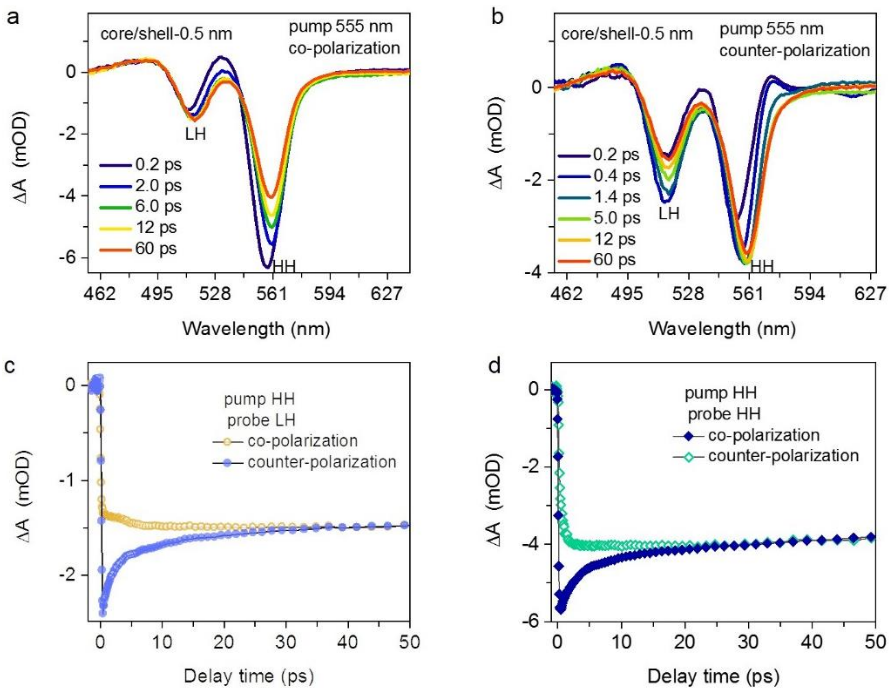

Figure S5. TA spectra of CdSe/ZnS core/shell-0.5 nm NPLs measured with (a) co- and (b) counter-polarized pump/probe configurations. TA kinetics of CdSe/ZnS NPLs measured with co- and counter-polarized pump/probe configurations probed at (c) HH (555 nm) and (d) LH (512 nm) exciton bleach features.

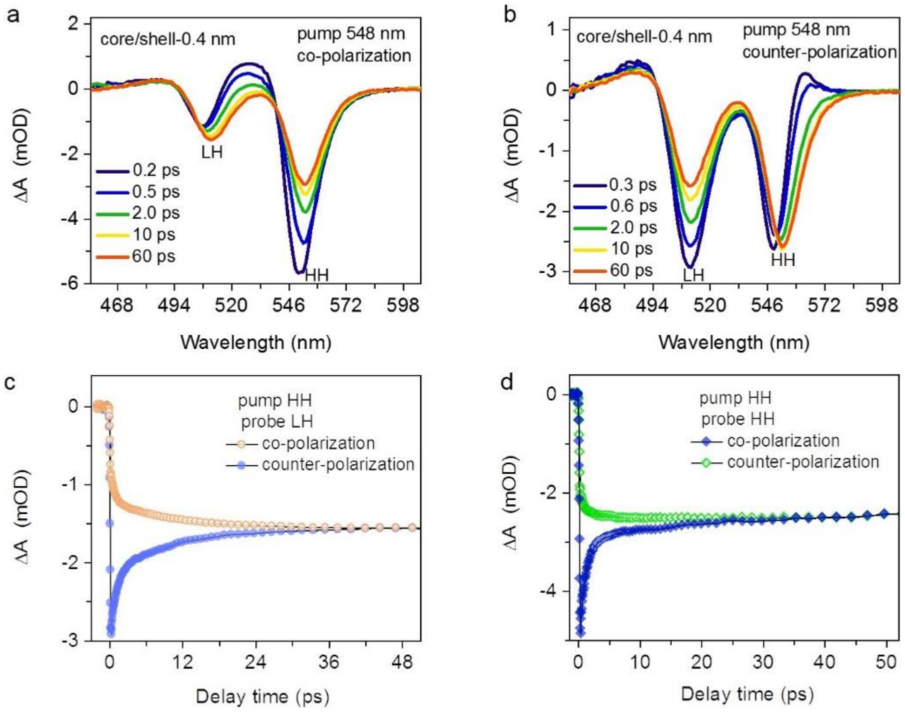

Figure S6. TA spectra of CdSe/ZnS core/shell-0.4 nm NPLs measured with (a) co- and (b) counter-polarized pump/probe configurations. TA kinetics of CdSe/ZnS NPLs measured with co- and counter-polarized pump/probe configurations probed at (c) HH (548 nm) and (d) LH (506 nm) exciton bleach features.

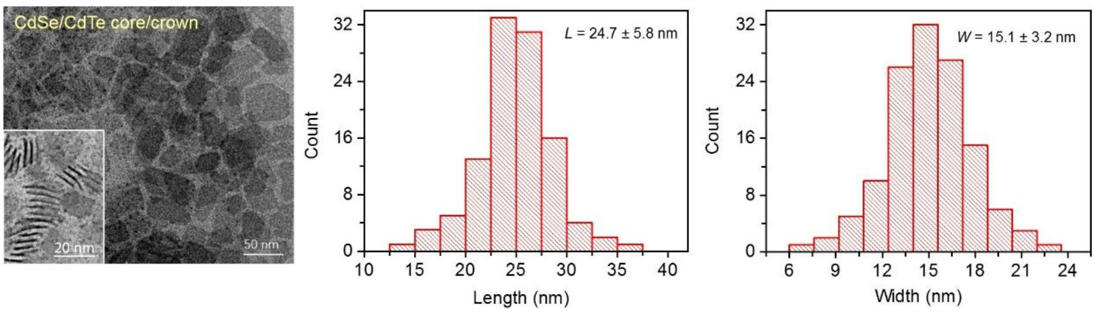

Figure S7. TEM images of CdSe/CdTe core/crown colloidal NPLs (left) and histograms for the NPLs' length (middle) and width (right).

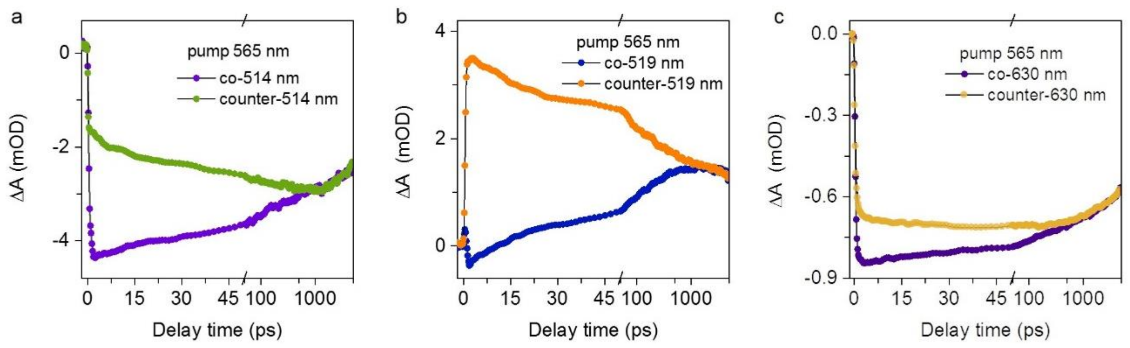

Figure S8. TA kinetics of CdSe/CdTe NPLs measured with co- and counter-polarized pump/probe configurations probed near the CdSe HH bleach at (a) 514 nm and (b) 519 nm, as well as (c) near the CT bleach center at 630 nm.

Estimation of exciton binding energy by fitting absorption spectra

We fit the absorption spectrum of CdSe/ZnS core/shell NPLs of varying shell thicknesses using a quantum well (QW) absorption model.[4, 5] The excitonic absorption of a QW exciton is:

$$\alpha_X(E) = \frac{1}{2\eta}\left[erf\left(\frac{E-E_0}{\gamma_X} - \frac{\gamma_X}{2\eta}\right)+1\right]\cdot \exp\left(\frac{\gamma_X{}^2}{4\eta^2} - \frac{E-E_0}{\eta}\right) \qquad \text{(S1)},$$

where E_0 is the absolute energy of the exciton, γ_X is the exciton line width and η is an asymmetric broadening factor. The corresponding absorption of the continuous band is:

$$\alpha_C(E) = \frac{H_C}{2}\left[erf\left(\frac{E-E_0-E_b}{\gamma_C}\right)+1\right] \qquad \text{(S2)},$$

where H_C is the step height of the continuum edge, γ_C is its width and E_b is the exciton binding energy. The absorption of the NPLs is the sum of excitonic and continuous absorptions of HH, LH and SO bands:

$$\alpha(E) = \sum_{i=HH,LH,SO} A_i\left(\alpha_{X,i}(E)+\alpha_{C,i}(E)\right) \qquad \text{(S3)}.$$

As explained in the main text, the the ratio between the E_b of LH and HH excitons is fixed at 1.51 in all the fits. The fitting parameters are listed in Table S1.

Table S1. Fitting parameters for absorption spectra of CdSe/ZnS core/shell NPLs with varying shell thicknesses.

**core/shell-1.5 nm**	**HH**	**LH**	**SO**
$\boldsymbol{E_b}$ (eV)	0.042	0.062	0.153
$\boldsymbol{E_0}$ (eV)	1.975	2.175	2.599
γ_X (eV)	0.049	0.068	0.216
$\boldsymbol{H_C}$ (eV)	9.22	6.21	25.3
γ_C (eV)	0.049	0.068	0.216
η	0.032	0.032	0.051
$\boldsymbol{A}$	0.194	0.212	0.181

**core/shell-1.1 nm**	**HH**	**LH**	**SO**
$\boldsymbol{E_b}$ (eV)	0.044	0.066	0.139
$\boldsymbol{E_0}$ (eV)	2.065	2.283	2.743
γ_X (eV)	0.039	0.058	0.132

$\boldsymbol{H}_C$ (eV)	8.38	5.55	19.83
γ_C (eV)	0.039	0.058	0.132
η	0.028	0.028	0.042
A	0.141	0.167	0.086

**core/shell-0.8 nm**	**HH**	**LH**	**SO**
$\boldsymbol{E}_b$ (eV)	0.048	0.072	0.168
$\boldsymbol{E}_0$ (eV)	2.116	2.319	2.803
γ_X (eV)	0.049	0.066	0.131
$\boldsymbol{H}_C$ (eV)	7.43	4.53	11.93
γ_C (eV)	0.049	0.066	0.131
η	0.031	0.031	0.034
A	0.107	0.126	0.099

**core/shell-0.5 nm**	**HH**	**LH**	**SO**
$\boldsymbol{E}_b$ (eV)	0.056	0.085	0.166
$\boldsymbol{E}_0$ (eV)	2.211	2.394	2.811

γ_X (eV)	0.052	0.071	0.128
H_C (eV)	6.44	3.64	9.23
γ_C (eV)	0.052	0.071	0.128
η	0.024	0.033	0.035
A	0.084	0.091	0.061

core/shell-0.4 nm	HH	LH	SO
E_b (eV)	0.061	0.092	0.152
E_0 (eV)	2.239	2.419	2.848
γ_X (eV)	0.057	0.072	0.128
H_C (eV)	6.41	3.38	10.01
γ_C (eV)	0.057	0.072	0.128
η	0.021	0.038	0.026
A	0.069	0.073	0.056

core only	HH	LH	SO

E_b (eV)	0.151	0.228	0.165
E_0 (eV)	2.403	2.541	2.888
γ_X (eV)	0.017	0.064	0.075
H_C (eV)	4.71	0.72	2.81
γ_C (eV)	0.017	0.064	0.075
η	0.056	0.028	0.118
A	0.054	0.031	0.027

Table S2. Fitting parameters for the electron spin polarization decay of CdSe/ZnS core/shell NPLs with varying shell thicknesses.

Sample	A1 (%)	τ_1 (ps)	A2 (%)	τ_2 (ps)	τ_{ave} (ps)
core/shell-1.5 nm	52.5	3.7	47.5	33.9	18.0
core/shell-1.1 nm	51.9	1.7	48.1	15.7	8.4
core/shell-0.8 nm	52.1	1.7	47.9	14.0	7.6
core/shell-0.5 nm	54.2	1.0	45.8	10.7	5.4
core/shell-0.4 nm	60.2	1.0	39.8	10.2	4.6

REFERENCES:

1. A. H. Khan, V. Pinchetti, I. Tanghe, Z. Dang, B. Martín-García, Z. Hens, D. Van Thourhout, P. Geiregat, S. Brovelli and I. Moreels, *Chem. Mat.*, **2019**, *31*, 1450-1459.

2. K. F. Wu, Q. Y. Li, Y. Y. Jia, J. R. McBride, Z. X. Xie and T. Q. Lian, *ACS Nano*, **2015**, *9*, 961-968.

3. Y. Altintas, U. Quliyeva, K. Gungor, O. Erdem, Y. Kelestemur, E. Mutlugun, M. V. Kovalenko and H. V. Demir, *Small*, **2019**, *15*, 1804854.

4. K. Leosson, J. R. Jensen, W. Langbein and J. M. Hvam, *Phys. Rev. B*, **2000**, *61*, 10322-10329.

5. J. Q. Grim, S. Christodoulou, F. Di Stasio, R. Krahne, R. Cingolani, L. Manna and I. Moreels, *Nat. Nanotechnol.*, **2014**, *9*, 891-895.
